# Lycium ruthenicum Murr. alleviates nonalcoholic fatty liver in mice

**DOI:** 10.1002/fsn3.1445

**Published:** 2020-03-18

**Authors:** Keke Lu, Jing Wang, Yueyuan Yu, Yikuan Wu, Zhao He

**Affiliations:** ^1^ Shandong Provincial Hospital and School of Medicine Shandong University Jinan China; ^2^ State Key Laboratory of Food Science and Technology School of Food Science and Technology Jiangnan University Wuxi China; ^3^ Shandong Key Laboratory of Endocrinology and Lipid Metabolism Shandong Provincial Hospital Jinan China; ^4^ Institute of Endocrinology and metabolism Shandong Academy of Clinical Medicine Jinan China

**Keywords:** antioxidant, cholesterol, *Lycium ruthenium* Murr. (LRM), nonalcoholic fatty liver, reactive oxidative species

## Abstract

Oxidative stress and inflammation contribute to hypertriglyceridemia‐induced nonalcoholic fatty liver disease (NAFLD). Cholesterol‐enriched diets increase the risk of NAFLD. *Lycium ruthenium* Murr. (LRM) contains water‐soluble antioxidant proanthocyanidins. Whether *Lycium ruthenium* Murr. improves NAFLD remains elusive. In this study, we established a model of NAFLD‐induced by cholesterol‐enriched high‐fat diet (western diet) in *ApoE* ^−/−^ mice; oxidative stress and inflammation were examined and intervened by supplement of *Lycium ruthenium* Murr. (LRM) extracts. LRM supplement did not influence body weight gain, food intake, and lipotoxicity of mice. LRM supplement significantly alleviated triglyceride accumulation in liver, with reduced inflammation, elevated GSH‐Px activity, and reduced MDA levels. The expression of fatty acids oxidative gene *Scd1* was significantly increased, and fatty acids synthesis‐related gene *Pparγ* was dramatically downregulated on mRNA level in liver of mice with LRM supplement. These data demonstrated that LRM supplement decreased ROS production and inflammation, increased fatty acids oxidation, and reduced fatty acids synthesis in liver, leading to ameliorate the development of NAFLD induced by high western diet. Thus, oxidative stress and inflammation also are involved in the pathogenesis of western diet‐induced NAFLD, which is independent of obesity.

## Graphical abstract

Nonalcoholic fatty liver disease (NAFLD) is too much fat stored in liver cells, which is mainly caused by excessive triglycerides or cholesterol, but no effective drugs or approach to treat this disease. Lycium ruthenium Murr. (LRM) contains water‐soluble antioxidant proanthocyanidins, which can efficiency improve NAFLD. Therefore, Lycium ruthenium Murr. is a health food for reducing the risk of NAFLD.

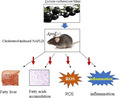

**Abbreviations**
LRM: *Lycium ruthenium* Murr
NAFLD: nonalcoholic fatty liver disease
ROS: Reactive oxidative species

## 1 INTRODUCTION

Nonalcoholic fatty liver disease (NAFLD) is a manifestation of metabolic syndrome, which covers a spectrum of liver changes, from steatosis to a complex pattern with hepatocellular injury and inflammation in the absence of alcohol intake (Bedossa, 2017; Bellentani, 2017). Fatty acid accumulation in the liver provokes multifaceted pathological processes of NAFLD. Cholesterol‐induced NAFLD increases the risk of cardiovascular disease (CVD) and is often associated with atherosclerosis (Kim et al., 2014). Currently, a “two‐hit hypothesis” is used to interpret the pathogenesis of NAFLD: The first hit is triglyceride accumulates within hepatocytes to induce simple steatosis and increases liver injury; the second hit is primary lipotoxicity caused by inflammation and oxidative stress within hepatocytes (Tu et al., 2017; Xu et al., 2015). However, although triglycerides accumulate in hepatocytes, they do not accumulate in the arterial wall.

Recently studies have showed that hypercholesterolemia increased the risk of NAFLD (Ma et al., 2008; Tous, Ferre, Camps, Riu, & Joven, 2005). In addition, because cholesterol can be accumulated in arteries and liver, NAFLD induced by cholesterol overload accompanies by atherosclerosis in arteries. However, it is unknown if cholesterol‐induced hepatic steatosis for further injury is similar to triglyceride‐induced fatty liver. Oxidative stress and inflammation are recognized as major causes of the pathogenesis of NAFLD in obese patients (Roskams et al., 2003; Serviddio, Bellanti, & Vendemiale, 2013). Inflammation also aggravated the hypercholesterolemia‐induced NAFLD progress (Kim et al., 2014). However, whether oxidative stress also contributes to the pathogenesis of cholesterol‐induced NAFLD remains uncertain.

*Lycium ruthenium* Murr (LRM), which belongs to the genus Lycium of family solanaceae, mainly contains water‐soluble antioxidant proanthocyanidins (Duan et al., 2015; Jiao, Song, Zhang, Gao, & Li, 2015; Wu, Lv, Wang, & Wang, 2016). Studies have shown that flavonoids have high activities of scavenging free radical and antioxidative (Duan et al., 2015; Wu et al., 2016; Zheng et al., 2011). Flavonoids and related compounds play immunomodulatory, anti‐inflammatory, and antioxidant roles, which positively improve therapeutic effects on cardiovascular disease and NAFLD (Bajalan, Mohammadi, Alaei, & Pirbalouti, 2016; El‐Haci et al., 2013; Li et al., 2009). However, whether LRM can ameliorate the pathogenesis of western diet‐induced NAFLD is unknown.

Here, we investigated the protective effect of LRM on the development of cholesterol‐enriched high‐fat diet‐induced NAFLD. Our results showed that LRM significantly reduced fatty acids accumulation due to increased oxidation and reduced synthesis, inflammation, and ROS production in hepatocytes, resulting in alleviating nonalcoholic fatty liver disease in *ApoE* ^−/−^ mice, which is independent of obesity. Our results provided an alternative choice for the treatment of NAFLD and a research model for the pathogenesis of NAFLD.

## 2 MATERIALS AND METHODS

### 2.1 Crude flavonoids extract and antioxidant activity assay

The fruit of *Lycium ruthenium* Murr. (LRM) was purchased from Qinghai. The fruit of *Lycium ruthenium* Murr. was oven‐dried at 50°C and subsequently crushed into powder. Then, the powder was suspended in 75% ethanol (60°C, 1:20 w/v) for 30 min to remove the fruit residues, protein, and polysaccharide sediment through filter paper (Lumeng, Bodzin, & Saltiel, 2007). All extraction solutions were concentrated with a rotary evaporator under 60°C, then were dissolved in distilled water. Total flavonoids content was determined by spectrophotometer (El‐Haci et al., 2013). Each sample (1 ml) was mixed with 0.5 ml of NaNO_2_ solution (5%). After 6 min, 0.5 ml of Al (NO_3_)_3_ solution (10%) was added into the mixture and allowed to stand for another 6 min. Then, 2 ml of NaOH solution (4%) was added to the mixture and stood for another 15 min. Absorbance of the mixture was determined at 510 nm versus water blank. A calibration curve was performed in parallel under the same operating conditions with rutin as a positive control. The sample was measured 3 times to obtain the average value. Results were presented as rutin equivalent per gram of dry extract (mg RU/100 g dry).

The effect of LRM on scavenging DPPH radical was determined by the modified method described as previous report (Li et al., 2009). Briefly, 2 ml of DPPH solution (2 mM dehydrated alcohol) was added to 1.0 ml of crude flavonoids in water. The mixture was shaken and stood for 30 min at room temperature in the dark. The absorbance was measured at 517 nm with a UV–vis spectrophotometer. The DPPH radical scavenging effect was calculated as follows: DPPH scavenging effect (%) = (A_0_ − (A − Ab))/A_0_ × 100%, where A_0_ is the A517 of DPPH without sample, A is the A517 of sample and DPPH, and Ab is the A517 of sample without DPPH.

Hydroxyl radical scavenging activity of the crude flavonoids from LRM was determined as previous report (Balavigneswaran, Kumar, Packiaraj, Veeraraj, & Prakash, 2013). Briefly, reaction mixture contained 1.0 ml of FeSO_4_, 1.0 ml of salicylic acid, 1.0 ml of flavonoids solutions at different concentrations (0.1–0.8 mg/ml), and 1.0 ml of 8.8 mM H_2_O_2_. Each was added sequentially, and the reaction mixture was incubated at 37°C for 30 min. The absorbance was recorded at 510 nm, and the scavenging activity of the flavonoids was calculated according to the following equation: Hydroxyl radical scavenging effect (%) = (A_0_ − (Ax‐Ax_0_))/A_0_ × 100%, where A_0_ is the A510 of control, Ax is the A510 of sample, and Ax_0_ is the A510 of sample without H_2_O_2_.

### 2.2 Animals and assays

All experimental procedures were performed in accordance with guidelines for Institutional Animal Care and approved by the Animal Ethics Committee of Jiangnan University 2015‐02 and Shandong Provincial Hospital. Male *ApoE* null mice (*ApoE* ^−/−^) on a C57BL/6 background were purchased from Model Animal Research Center of Nanjing University (MARC). Western Diet was purchased from Research Diets Co., LTD (21% fat and 1.25% cholesterol, D12079B, Table 1). At the age of 5‐week, *ApoE* ^−/−^ mice were randomly separated into three groups (*n* = 10 per group). The control group was fed on normal control diet (NC group); mice in the other two groups were fed a Western Diet (WD) for 12 weeks. After 8 weeks on a WD, mice were administered with flavonoids extracts (140 mg kg day^‐1^, LRM group) and distilled water (WD) by oral gavages. Body weight was scaled once a week, and food intake was measured three times a week. Oil red O was purchased from Sigma‐Aldrich. H&E and oil red O staining were performed as previous report (Kennedy et al., 2005; Sun et al., 2016).

**Table 1:** D12079B, RD Western Diet composition sheet

Product #	D12079B		98,121,701	
%	gm	kcal	gm	kcal
Protein	20	17	17	17
Carbohydrate	50	43	71	73
Fat	21	40	4	10
Total		100		100
kcal/gm	4.68		3.91	
*Ingredient*				
Casein, 80 Mesh	195	780	195	780
DL‐Methionine	3	12	3	12
Corn Starch	50	200	404.4	1617.6
Maltodextrin 10	100	400	100	400
Sucrose	341	1,364	341	1,364
Cellulose, BW200	50	0	50	0
Milk Fat, Anhydrous	200	1,800	0	0
Corn Oil	10	90	52.5	472.5
Ethoxyquin	0.04	0	0.04	0
Mineral Mix S10001	35	0	35	0
Calcium Carbonate	4	0	4	0
Vitamin Mix V10001	10	40	10	40
Choline Bitartrate	2	0	2	0
Cholesterol	1.5	0	0	0
FD&C Yellow Dye #5	0	0	0.05	0
FD&C Blue Dye #1	0	0	0.05	0
FD&C Red Dye #40	0	0	0	0
Total	1,001.54	4,686	1,197.04	4,686.1

Serum total cholesterol (TC), triacylglycerol (TG), low‐density lipoprotein cholesterol (LDL‐c), high‐density lipoprotein cholesterol (HDL‐c), aspartate aminotransferase (AST), and alanine aminotransferase were measured by an automated chemistry analyzer (Roche Modular P800; Roche) (Lumeng et al., 2007). The amount of TBA reactive substances as malondialdehyde (MDA) and GSH‐Px activities in the liver tissue was measured according to the manufacturer's instructions. The total protein was assessed with Bradford reagent at 562 nm, and bovine serum albumin was used as the standard for the analyses. The change in the absorbance at 532 and 412 nm was monitored by a spectrophotometer. One unit of GSH‐Px was defined as micromoles of nicotinamide adenine dinucleotidephosphate‐oxidase (NADPH) oxidized per minute. The activity was recorded in units/mg, and the MDA results were expressed as nmol/mg.

### 2.3 Gene expression

Tissues were homogenized in Trizol reagent (Invitrogen) for RNA isolation according to the manufacturer's instruction. cDNA was synthesized by using PrimeScript^®^ RT Master Mix Perfect Real Time (Takara). RT‐PCR was performed in triplicate with Bio‐Rad, iTaq TM Universal SYBR ^®^Green Supermix according to the manufacturer's protocol. QRT‐PCR was performed and analyzed on the QX200 Droplet Digital PCR system (Bio‐Rad). Primers were used: *Tnf‐α*: 5′‐CTCAGATCATCTTCTCAAAATTCGAGTGACA‐3′, 5′‐CTTCACAGAGCAATGACTCCAAAGT‐3′; *Il‐6*:5′‐CTTCCATCCAGTTGCCTTCTTG‐3′, 5′‐AATTAAGCCTCCGACTTGTGAAG‐3′; *Il‐4*:5′‐CCAACTGCTTCCCCCTCTG‐3′, 5′‐TCTGTTACGGTCAACTCGGTG‐3′; *Il‐10*:5′‐GACTTTAAGGGTTACCTGGTGG‐3′, 5′‐CACATGCGCCTTGATGTCTG‐3′; *Ppar‐γ*: 5′‐TGGAATTAGATGACAGCGACTTGG‐3′, 5′‐CTGGAGCAGCTTGGCAAACA‐3′; *Srebp‐1c*: 5′‐GAGCGAGCGTTGAACTGTAT‐3′, 5′‐ ATGCTGGAGCTGACAGAGAA‐3′; *Fasn*: 5′‐TGTGAGTGGTTCAGAGGCAT‐3′, 5′‐TTCTGTAGTGCCAGCAAGCT‐3′; *β‐actin*: 5′‐ CCCAGGCATTGCTGACAGG‐3′, 5′‐TGGAAGGTGGACAGTGAGGC‐3′.

### 2.4 Statistical analysis

All data were presented as mean ± *SEM*, and differences were assessed by Tukey's multiple comparisons after analysis of variance (ANOVA). Statistical significance was defined as *p < .05. **p < .01.

## 3 RESULTS

### 3.1 *Lycium ruthenium* Murr. extracts have strong scavenging free radical activity

**Figure 1:**
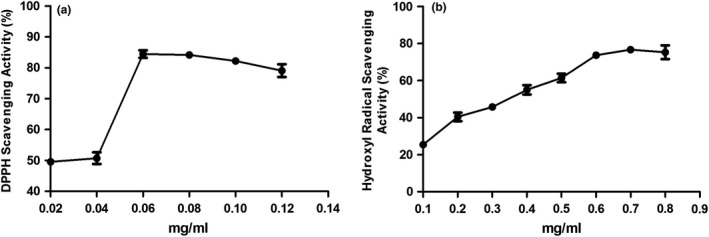
Antioxidative activity of LRM extracts. (a) Scavenging effects of LRM extracts on DPPH radical; (b) Scavenging effects of LRM extracts on hydroxyl radical

Since flavonoids are natural antioxidant with strong free radical scavenging activity, it promoted us to examine the antioxidant activities of LRM extracts by the DPPH assay which is widely used to evaluate the free radical scavenging activity of plant extraction. As shown in Figure 1a, the scavenging activity of DPPH radical was significantly enhanced with increasing concentration of extracts and reached a peak when the concentration was more than 0.06 mg/ml. Additionally, the antioxidant activity of substance is also determined by scavenging the hydroxyl free radical. As shown in Figure 1b, LRM extracts exhibited high hydroxyl radical scavenging activities in a dose dependent manner. Taken together, these observations showed that LRM extracts have strong free radical scavenging and antioxidative activity.

### 3.2 Mice body weight gain remains unchanged with LRM supplement

**Figure 2:**
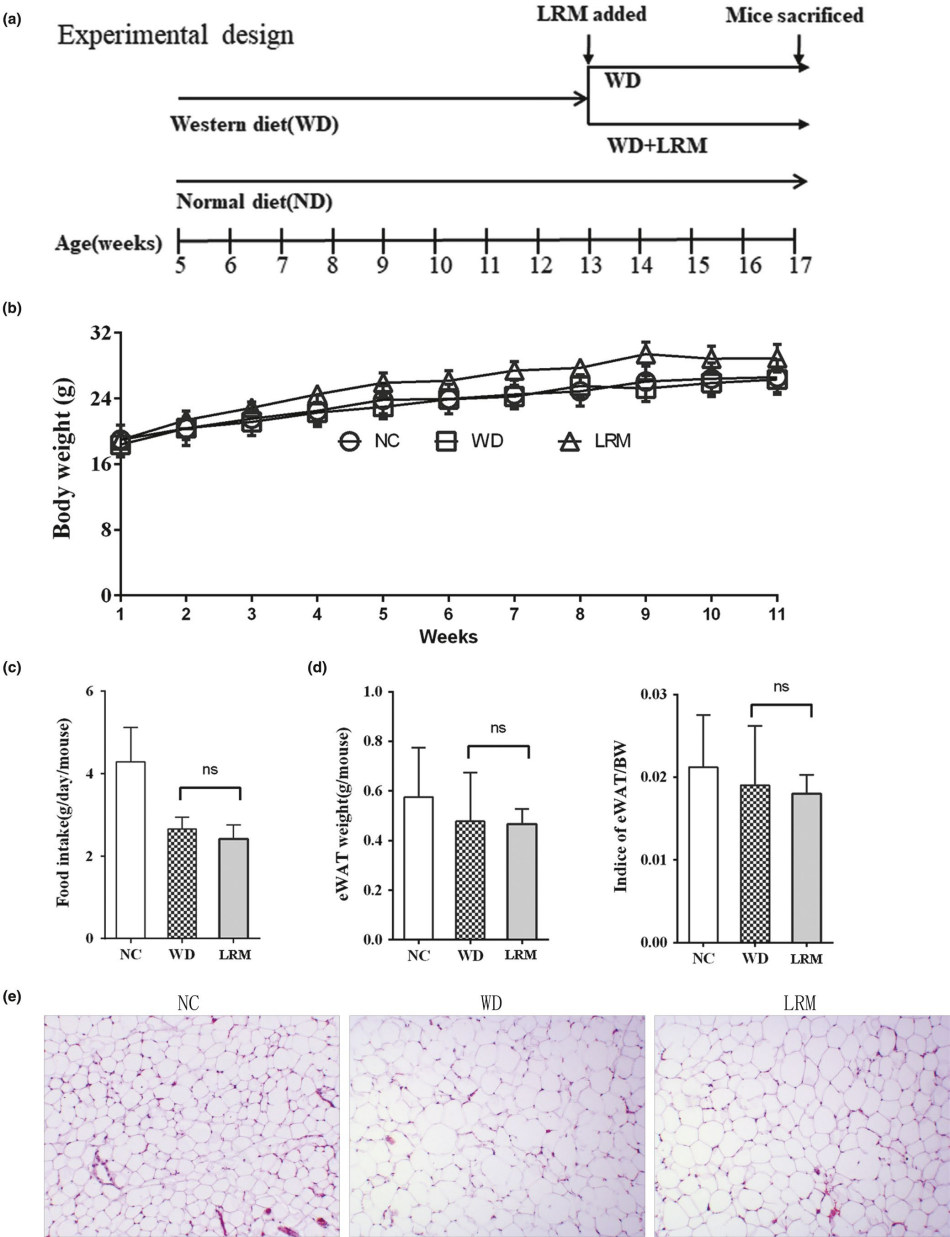
Body weight. (a) experimental design strategy; (b) Body weight changes during the experimental 12 weeks. Body weight gains from 6 to 17 weeks; (c) Average daily food intake from 6 to 17 weeks; (d) eWAT and indices of eWAT/body weight; (e) white fat tissue H&E staining. NC: normal control; WD: cholesterol‐enriched western diet; LRM: extracts of *Lycium ruthenicum* Murr

To investigate the effect of LRM on the pathogenesis of NAFLD, 5 weeks male *ApoE* ^−^ *^/^* ^−^ mice were fed on western diet (WD) or normal diet (ND) for 12 weeks. After 8 weeks on WD, LRM was administered to half of mice fed on WD for another 4 weeks (Figure 2a). The body weight and food intake of mice were monitored. After 12 weeks, body weight was similar in WD‐ and ND‐fed mice, indicating that cholesterol‐enriched low‐fat diet did not change the body weight compared with normal low‐fat diet. Food daily intake and body weight gain were not significantly changed in WD‐fed mice with LRM supplement compared with WD‐fed mice without LRM supplement (Figure 2b and c). Consistently, the epididymal fat pad weight was similar in mice with or without LRM supplement (Figure 2d). Mice on WD with LRM supplement had similarly size of adipocyte compared with mice on WD (Figure 2e). Together, these observations demonstrated that LRM supplement did not change the state of energy balance.

### 3.3 LRM supplement ameliorates the development of NAFLD

**Figure 3:**
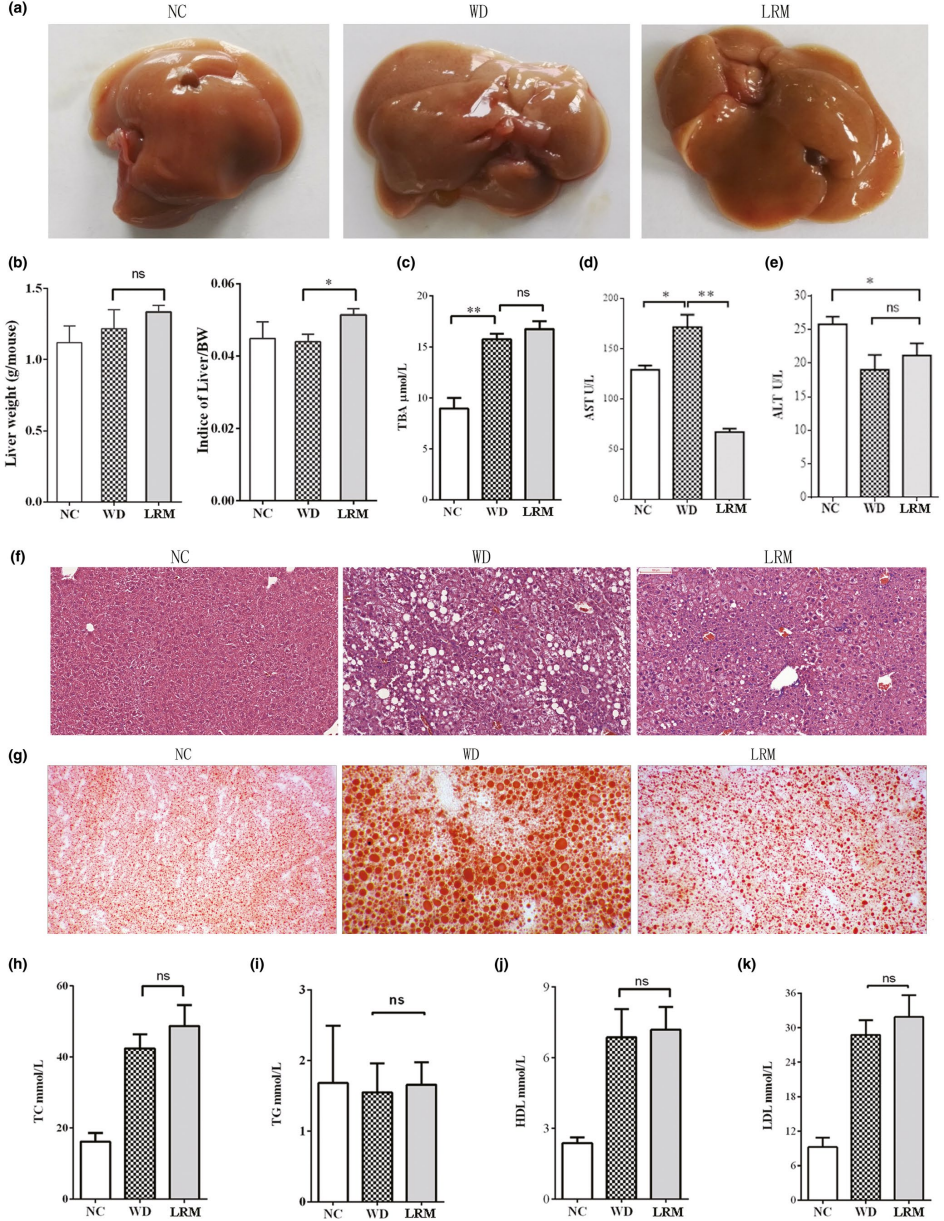
Hepatic steatosis and lipids. (a) Representative morphology of liver; (b) liver weight and indices of liver/body weight; (c) Total Bile Acid (TBA) content; (d) AST level; (e) ALT level; (f) hematoxylin and eosin staining of liver section; (g) Representative image of liver by Oil red O staining; (h) TC levels; (i) TG levels; (j) HDL levels; (k) LDL levels; TC: total cholestenone; TG: total glycerin; HDL: high‐density lipoprotein; LDL: low‐density lipoprotein. Scale bars 200 μm in H&E staining and Oil red O staining. NC: normal control; WD: western diet; LRM: extracts of *Lycium ruthenicum* Murr

The liver morphology, weight, and indices of liver/body weight were similar in mice on WD with LRM supplement and mice on WD (Figure 3a and b). Cholesterol‐enriched diet dramatically increased total bile acid (TBA) level in mice compared with NC (Figure 3c). However, TBA level was similar in mice on WD with and without LRM supplement (Figure 3c). Serum AST levels in the mice on WD were significantly elevated compared with mice fed on NC, while LRM supplement significantly reduced AST levels (Figure 3d), suggesting LRM protected liver injury. However, serum ALT level was similar between WD and LRM group (Figure 3e). Consistently, mice on WD developed severe hepatic steatosis compared with mice on NC, revealed by H&E and oil red O staining (Figure 3f and g). LRM supplement significantly decreased the size of fat droplet in liver of mice on WD, indicating a protective role of LRM in cholesterol‐induced NAFLD (Figure 3g). Hypercholesterolemia was induced in *ApoE* ^−^ *^/^* ^−^ mice fed on cholesterol‐enriched diet for 12 weeks (Figure 3h). The levels of serum TC, HDL‐c and LDL‐c were significantly increased in mice on WD compared with mice on NC (Figure 3h, j and k), indicating a lipotoxicity status. However, mice on WD with LRM supplement displayed similar TC, TG, LDL, and HDL‐c levels compared to mice on WD without LRM supplement (Figure 3h–k), indicating dyslipidemia was unchanged by LRM supplement. These observations suggested that LRM supplement alleviated the pathogenesis of cholesterol‐enriched diet‐induced NAFLD, independent of lipotoxicity state.

### 3.4 LRM Increases fatty acid oxidation and reduces fatty acid synthesis

**Figure 4:**
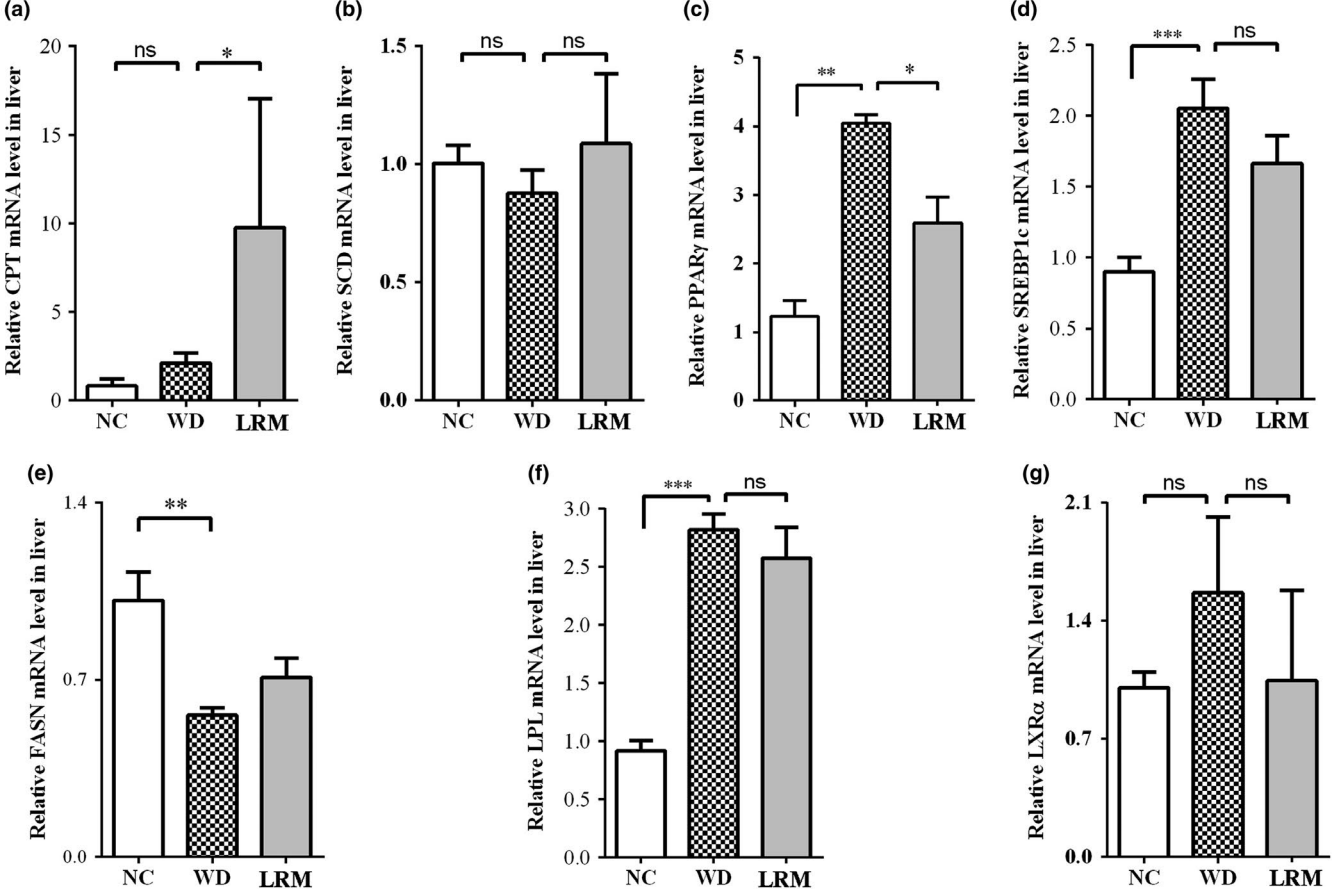
Relative expression of lipogenesis and lipolysis genes. (a) relative level of *Cpt* mRNA; (b) relative level of *Scd* mRNA; (c) relative level of *Pparγ* mRNA; (d) relative level of *Srebp1c* mRNA; (e) relative level of *Fasn* mRNA; (f) relative level of *Lpl* mRNA; (g) relative level of *Lxrα* mRNA

Excessive fat accumulation in liver leads to NAFLD, lipogenesis, fatty acid oxidation, and transportation are important ways to regulate the development of hepatic steatosis. As shown in Figure 4, LRM supplement significantly enhanced oxidation gene *Cpt* mRNA levels compared with mice on WD (Figure 4a), and *Scd* mRNA level was also similar in mice on WD with or without LRM supplement (Figure 4b), indicating elevated fatty acid oxidation in mice with LRM supplement. The expression of *Pparγ* and *Srebp1* genes, which were associated with fat acid synthesis, was decreased in mice on WD with LRM supplement compared with mice on WD (Figure 4c‐e). LRM supplement decreased *Pparγ* and *Fasn* mRNA levels in mice, indicating reduced fatty acid synthesis in liver. In lipids transportation, *Lpl* mRNA level showed significant increase in mice on WD compared with ND (Figure 4f), indicating high cholesterol diet promoted lipid transportation. However, no significant change of *Lpl* mRNA level was observed in mice with or without LRM supplement (Figure 4f), suggesting that LRM did not regulate lipid transportation. Liver X receptor alpha is an important regulator of cholesterol homeostasis. *Lxrα* mRNA level was not significantly changed in mice with or without LRM supplement (Figure 4g), indicating that LRM supplement did not significantly affect cholesterol homeostasis. Together, these results demonstrated that LRM supplement enhanced lipid oxidation and reduced fatty acid synthesis in liver.

### 3.5 LRM reduces inflammation and ROS production

**Figure 5:**
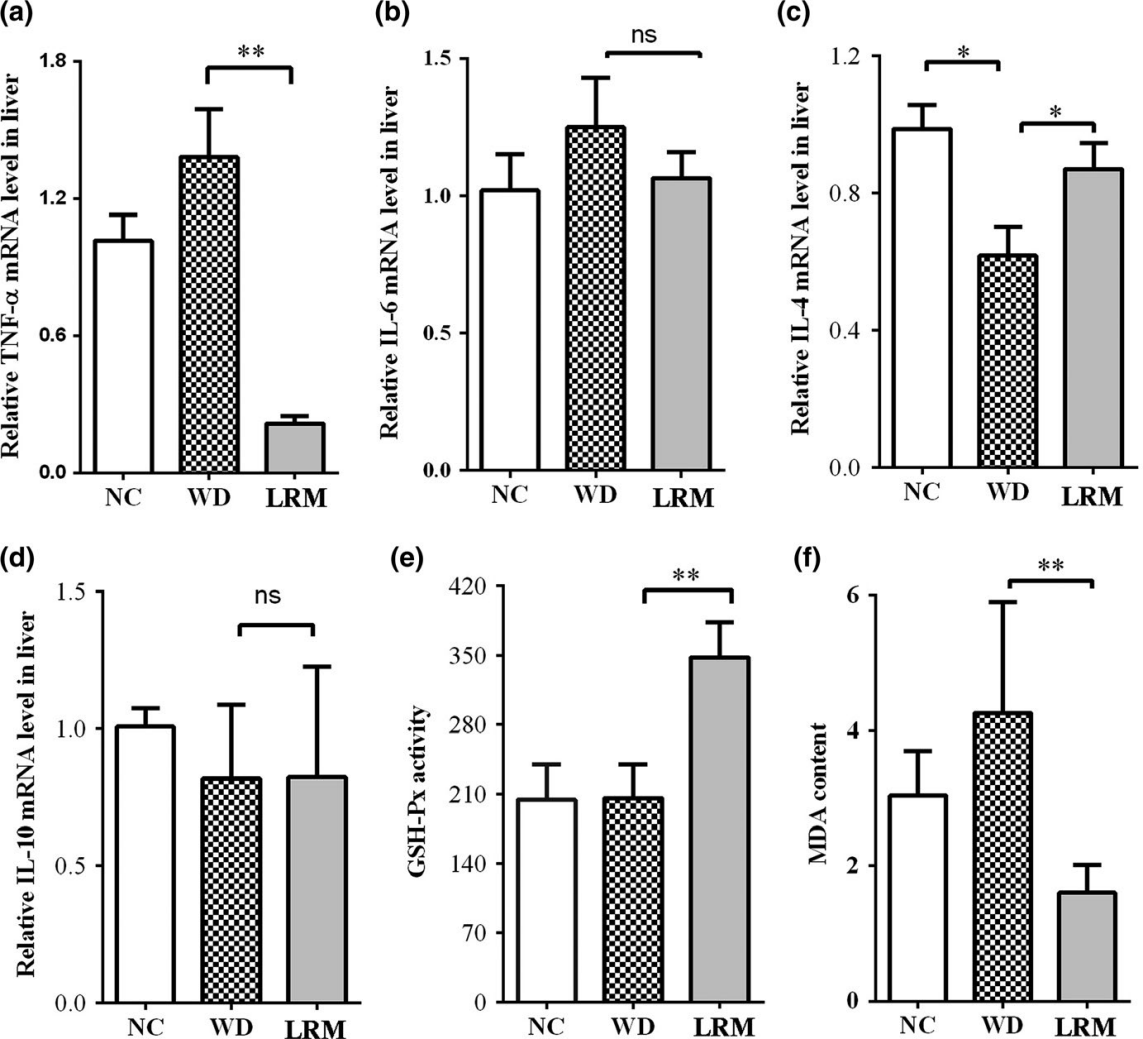
inflammatory genes expression and ROS. (a) *Tnf‐α*; (b) *Il‐6*; (c) *Il‐4*; (d) *Il‐10* mRNA levels in liver tissues. (e) GSH‐Px activity in liver tissue; (f) MDA content in liver tissue. NC: normal control; WD: western diet; LRM: extracts of *Lycium ruthenicum* Murr

Chronic inflammation is associated with the progression of NAFLD toward higher risk cirrhotic states (Wijesundera et al., 2016). To investigate whether LRM alleviated the pathogenesis of NAFLD by reducing inflammation, we first examined the expression of pro‐inflammatory genes. The mRNA level of *Tnf‐α* was remarkably decreased in mice with LRM supplement, indicating an anti‐inflammatory effect of LRM (Figure 5a). However, the expression of pro‐inflammatory gene *Il‐6* in liver was not significantly changed by LRM supplement (Figure 5b). The transcript level of anti‐inflammatory *Il‐4* gene was dramatically increased in mice on WD with LRM supplement compared with WD‐fed mice (Figure 5c), while *Il‐10* mRNA level was similar in mice with or without LRM supplement (Figure 5d). These results indicated that LRM supplement improved inflammation by regulating *Tnf‐α* and *Il‐4* expression.

Reactive oxidative species (ROS) are strongly associated with the progress of hepatic steatosis (Videla et al., 2004). To address this, we first measured the GSH‐Px activities and MDA levels, which were responsible for oxidative stress in tissue. We found that mice with LRM supplement showed dramatically elevated GSH‐Px activities compared with mice on WD (Figure 5e). MDA levels in liver were significantly higher in mice on WD than NC. LRM supplement dramatically reduced MDA levels in mice (Figure 5f), indicating an inhibitory effect of LRM on ROS production. Together, these observations suggested that LRM supplement reduced oxidative stress induced by cholesterol‐enriched high‐fat diet.

## 4 DISCUSSION

In the study, we found that oxidative stress and inflammation are associated with cholesterol‐induced NAFLD in *ApoE* ^−^ *^/^* ^−^ mice. Antioxidant LRM supplement remarkably slowed down the process of NAFLD induced by cholesterol‐enriched high‐fat diet, and reduced lipid deposition, inflammatory level and ROS production in liver. Our results demonstrated that LRM supplement ameliorated NAFLD development induced by cholesterol‐enriched high‐fat diet, which was independent of obesity (Pang et al., 2015).

Mice with LRM supplement had less fat deposition, coupled with fewer and smaller vacuoles and lipid droplets in the liver. And the morphology of hepatic cells was similar to normal phenotype with mild hepatic steatosis and occasionally inflammatory cell infiltration. The lipogenesis gene was significantly suppressed and fatty acid oxidation gene was dramatically increased in mice with LRM supplement. Body weight gain of mice fed on WD for 12 weeks was similar to mice fed on control diet, indicating no obesity development. Consistently with previous studies that fat accumulation in the liver was independent of body mass index and intra‐abdominal and overall obesity (Seppala‐Lindroos et al., 2002).

Studies have indicated that systemic inflammation was strongly associated with the progress of hepatic steatosis in obese individuals (Alisi et al., 2017; Lumeng et al., 2007). Inflammatory genes were activated in the liver and their expression were increased in NAFLD patients (Duan et al., 2015; Tarantino, Colicchio, et al., 2009; Tarantino, Conca, et al., 2009). During the progression of NAFLD, fatty acid accumulation in the liver activates the reactive oxygen species (ROS) and induces oxidative stress. These ROS, in turn, promotes lipid peroxidation and triggers TNF‐α‐regulated liver damage (Mari et al., 2006). Oxidant‐sensitive transcription factors such as nuclear factor‐κB (NF‐κB) are then also invigorated by ROS, upregulating the expression of cytokines including interleukin‐6 (IL‐6) (Stojsavljevic, Gomercic Palcic, Virovic Jukic, Smircic Duvnjak, & Duvnjak, 2014; Tarantino, Colicchio, et al., 2009; Tarantino, Conca, et al., 2009). Indeed, LRM supplement downregulated the *Tnf‐α* mRNA levels in liver (Figure 3). The GSH‐Px activities were significantly upregulated in mice with LRM supplement, coupled with lower MDA level in liver tissue, indicating an inhibitory effect of LRM on ROS production (Figure 4). All these results revealed that LRM ameliorates the pathogenesis of hepatic steatosis induced by cholesterol‐enriched high‐fat diet, coupled with reduced ROS production and inflammatory levels.

In conclusion, our results indicated that oxidative and inflammation are involved in the pathogenesis of cholesterol‐enriched high‐fat diet‐induced NAFLD, which is independent of obesity. Reduction of oxidative stress and inflammation by antioxidant LRM suppressed the progression of NAFLD‐induced by cholesterol‐enriched high‐fat diet. However, the really active components in the crude flavonoids still need to be identified by further experiments. Further experiments are also required for the elucidation of the underlying mechanism of LRM ameliorating the pathogenesis of hepatic steatosis.

## CONFLICT OF INTEREST

The authors declare that they do not have any conflict of interest.

## AUTHOR CONTRIBUTIONS

Yueyuan Yu and Zhao He designed the research; Yueyuan Yu performed research; Keke Lu, Jing Wang, Yueyuan Yu, Yikuan Wu, and Zhao He analyzed data; Keke Lu, Yueyuan Yu, and Zhao He wrote the paper.

## ETHICAL APPROVAL

This study does not involve any human testing, and the animal study's protocols and procedures were ethically reviewed and approved by the Jiangnan University.

## INFORMED CONSENT

Written informed consent was obtained from all study participants.
